# The Effect of Particle Shape on the Compaction of Realistic Non-Spherical Particles—A Multi-Contact DEM Study

**DOI:** 10.3390/pharmaceutics15030909

**Published:** 2023-03-10

**Authors:** Kostas Giannis, Arno Kwade, Jan Henrik Finke, Carsten Schilde

**Affiliations:** 1 Center of Pharmaceutical Engineering (PVZ), Technische Universität Braunschweig, Franz-Liszt-Str. 35A, 38106 Braunschweig, Germany; 2 Institute for Particle Technology (iPAT), Technische Universität Braunschweig, Volkmaroder Str. 5, 38104 Braunschweig, Germany

**Keywords:** large deformation, FEM, DEM, plastic deformation, compaction, particle shape, morphology

## Abstract

The purpose of this study was to investigate the deformation behavior of non-spherical particles during high-load compaction using the multi-contact discrete element method (MC-DEM). To account for non-spherical particles, the bonded multi-sphere method (BMS), which incorporates intragranular bonds between particles, and the conventional multi-sphere (CMS), where overlaps between particles are allowed to form a rigid body, were used. Several test cases were performed to justify the conclusions of this study. The bonded multi-sphere method was first employed to study the compression of a single rubber sphere. This method’s ability to naturally handle large elastic deformations is demonstrated by its agreement with experimental data. This result was validated further through detailed finite element simulations (multiple particle finite element method (MPFEM)). Furthermore, the conventional multi-sphere (CMS) approach, in which overlaps between particles are allowed to form a rigid body, was used for the same objective, and revealed the limitations of this method in successfully capturing the compression behavior of a single rubber sphere. Finally, the uniaxial compaction of a microcrystalline cellulose-grade material, Avicel^®^ PH 200 (FMC BioPolymer, Philadelphia, PA, USA), subjected to high confining conditions was studied using the BMS method. A series of simulation results was obtained with realistic non-spherical particles and compared with the experimental data. For a system composed of non-spherical particles, the multi-contact DEM showed very good agreement with experimental data.

## 1. Introduction

Granular materials are unique in that they can behave as either solids or liquids. Sand, for instance, is composed of dissipative grains that interact with one another via repulsive and frictional contact forces. Despite their simplicity, the physics of granular materials is still poorly understood, leaving many open questions in a variety of fields, such as physics, process engineering, material science and geotechnical engineering. Cundall and Strack [1] pioneered the discrete element method (DEM) to model their physical behavior. The DEM numerically represents granular materials as a collection of particles rather than a continuum, and the bulk behavior of granular materials is determined by collective interactions among individual particles.

However, modeling the mechanical behavior of granular assemblies when subjected to high loads, such as mineral processing [2], soil compaction [3], and pharmaceutical tablets [4], is a difficult issue strongly affected by the shape of the individual particles. In reality, and in the vast majority of applications, the particle shape is non-spherical, but it may be reformed into a sphere by the spheronization [5] process. Due to the time required for contact detection and resolution, particle shape consideration is a computationally costly operation in DEM; hence, an ideal spherical particle approximation is frequently used as a fast alternative. Adjusting the rolling friction parameters to mimic the particle shape effects is one way to compensate for the lack of accuracy that is caused by ideal spherical representations [6]. However, this approach still does not allow for a thorough investigation of the impact of particle shape. 

Nonetheless, general agreement on the need for sophisticated particle shape representations has resulted in techniques that allow for shape consideration within DEM. Approaches that are now available include sphere clusters, superquadrics, and polyhedra. Two distinct approaches may be found in sphere clusters: (a) the multi-sphere (hereinafter referred to as the conventional multi-sphere) and (b) the bonded multi-sphere [7,8]. In the conventional multi-sphere (CMS) [9,10], many particles are “linked together” by overlapping particles experiencing multiple contacts [11] to resemble a rigid, unbreakable non-spherical particle. The bonded multi-sphere approach (BMS) [12], on the other hand, uses non-overlapping particles that are “touching” each other and bonded together with intragranular bonds to represent arbitrary particle shapes. The bonded multi-sphere model, which was first developed to simulate fracture initiation and evolution across mineral grains in rock [12], may also be used to approximate individual particles with complex shapes. The main advantage of this approach is that, by adjusting the bond parameters, the particles may behave both rigidly and deformably, and their breaking behavior can be simulated [8]. In contrast, ellipsoids and other quadric shapes can be constructed by employing superquadrics [13] and adjusting the shape parameters in the mathematical formulation. A comparison [10] of the conventional multi-sphere approach with superquadrics shows that using superquadrics saves computation time when simulating non-spherical particles, particularly blocky particles. Polyhedral particle models allow for the use of sharp corners and edges that may be used to create a variety of random particles [14]. Polyhedral particles can be used in a variety of applications [15,16], including crushing [17]. Nevertheless, these techniques have the problem of high computing costs, especially when compared to the use of solely spherical particles. For example, the neighbor search and contact resolution processing time that is necessary to investigate the interactions of thousands of non-spherical particles may render the study impractical. This limitation may arise when large numbers of particles need to be simulated.

The objective of this study was to investigate the impact of the particle shape on compaction profiles using the MC-DEM method. Due to computational time constraints, the simulations were limited to a small number of particles. The accuracy of the MC-DEM method had previously been validated by demonstrating its ability to model the compressibility properties of two pharmaceutical materials (Avicel^®^ PH 200 (FMC BioPolymer) and Pharmacel^®^ 102 (DFE Pharma)), assuming that the particles were spherical [4]. This study aimed to extend the MC-DEM method to include realistic non-spherical particles by combining it with BMS. The outcomes of both spherical and non-spherical particle simulations were compared to experimental data.

## 2. Theory

The particle simulations were performed using DEM. In this approach, Newton’s equations of motion were used to determine the relationship between the particle motion and forces acting on each particle. The translational and rotational motion equations of a particle are as follows:

(1) $$m_{i}{\ddot{a}}_{i}=\underset{j}{\sum }\left(F_{nij}+F_{tij}\right)+m_{i}g\;\;\;and\;\;I_{i}{\dot{\omega }}_{i}=\tau_{ij}$$

where $m_{i}$, ${\ddot{a}}_{i}$,$\;I_{i}$, and ${\dot{\omega }}_{i}$ are the mass, acceleration, moment of inertia, and angular velocity for particle i, respectively; $F_{nij}$, $F_{tij}$, and $\tau_{ij}$ are the normal force, tangential force, and torque acting on particles *i* and *j* at the contact points, respectively; g is the acceleration due to gravity.

In order to describe the force–displacement laws at the contact points, various contact models can be used. In general, and considering recent developments, contact laws may be classified into two distinct groups: (a) local contact models and (b) non-local contact models. While the contact is resolved locally in the first group [18,19], a non-local resolution based on the neighboring contact interaction is included in the second group [20,21,22].

In this study, an open-source code, LIGGGHTS [23,24], was used to perform numerical simulations. The force and torque contributions of particles can be calculated from three distinct contact models: normal contact, tangential sliding, and tangential rolling.

### 2.1. Hertz–Mindlin Contact Model

The most common contact model is the linear spring–dashpot model [1], in which the spring stiffness is assumed to be constant, and the Hertz contact model calculated using the Hertz theory [25] (e.g., nonlinear spring–dashpot model). For the accurate estimation of particle deformation, we related the interaction force to the overlap of two particles. It should be noted that the evaluation of interparticle forces based on overlap may not be sufficient to account for the inhomogeneous stress distribution inside the particles. The normal repulsive contact force is

(2) $$F_{n}=k_{n}\delta_{n}^{3/2}+\gamma_{n}\;{\dot{\delta }}_{n}$$

where $k_{n}=\frac{4}{3}E^{*}\sqrt{R^{*}}$ is the normal stiffness coefficient, $R^{*}=\frac{R_{i}R_{j}}{R_{i}+R_{j}}$ and $E^{*}=\frac{1-\nu_{i}^{2}}{E_{i}}+\frac{1-\nu_{j}^{2}}{E_{j}}$ are the effective radius and the effective Young’s modulus, and $R_{i},\;R_{j},\;\nu_{i},\;\nu_{j},\;E_{i},\;E_{j}$ are the radius, Poisson’s ratio, and Young’s modulus of particle i and j, respectively. $\delta_{n}$ is the normal overlap, ${\dot{\delta }}_{n}$ is the relative velocity in the normal direction of interacting particles, and $\gamma_{n}$ the viscoelastic damping constant for the normal contact viscosity. The tangential force is [20]

(3) $$F_{t}=k_{t}\delta_{t}^{3/2}+\gamma_{t}\;{\dot{\delta }}_{t}$$

where $k_{t}=8G^{*}\sqrt{R^{*}}$ and $G^{*}=\frac{2-\nu_{i}}{G_{i}}+\frac{2-\nu_{j}}{G_{j}}$ are the tangential stiffness coefficient and the effective shear modulus, respectively, and $G_{i},\;G_{j}$ are the shear moduli of particles i and j. $\delta_{t}$ is the tangential overlap, ${\dot{\delta }}_{t}$ is the relative velocity in the tangential direction of interacting particles, and $\gamma_{t}$ is the viscoelastic damping constant for tangential contact viscosity.

The tangential overlap, $\delta_{t}$, between particles obtained by an integrating surface and the relative tangential velocities during the elastic deformation of the contact is given as

(4) $$\delta_{t}=\int_{t}^{t+\Delta t}v^{t}dt\to \;\delta_{t}\approx v^{t}dt$$

where $v^{t}$ is the velocity component that is tangential to the contact surface and Δt is the timestep.

Through Coulomb’s law, the tangential force was connected to the normal force, $F_{t}\leq \mu F_{n}$, in the event of sliding, and the dynamic friction was $F_{t}=\mu F_{n}$. The dynamic and static friction coefficients can be assumed to be equal in this study: $\mu =\mu_{d}=\mu_{s}$. Due to the activated Coulomb friction, the static state necessitates the use of an elastic spring to provide a restoring force, i.e., a non-zero residual tangential force in the static equilibrium. By applying torque to the contacting surfaces, the rolling friction can be controlled. The constant directional torque (CDT) of the rolling friction, $\tau_{ij}$, used in this investigation is given by [4]

(5) $$\tau_{ij}=-\frac{\omega_{rel}}{\left|\omega_{rel}\right|}\mu_{r}R^{*}F_{n}$$

where $\omega_{rel\;}$=$\;\omega_{i}-\omega_{j}$, and $\omega_{i},\;\;\omega_{j}$ are the angular velocities of particles i and j, while $\mu_{r}$ is the rolling friction coefficient. The particles in these models are assumed to be spherical and without any deformation (simply pseudo-deformation) during simulation. Furthermore, particle forces are resolved locally because they comprise binary interactions between interacting particles.

### 2.2. Multi-Contact Adhesive Elastic–Plastic Model

Plastic deformation and particle shape changes are prominent in confined systems, such as tableting, where the powder is compressed under high loads. As a result, advanced nonlinear contact models that take both elastic–plastic contact deformation and adhesion into consideration are required. A multi-contact model recently developed by Giannis et al. [4] was employed in this study. This contact model differs from others since it employs a multi-contact technique to account for non-local contact forces.

The trace of the average stress tensor, along with Poisson’s ratio (ν), the contact area (A) between the interacting particles, and a material-dependent prefactor (β), have been used to account for multiple contacts acting on a particle.

The adhesive plastic force is

(6) $$F_{n}=\left\{ \begin{array}{ll} F_{0}+k_{1}\delta_{n}^{3/2}+\left(\beta vA_{ij}\right)P_{ij} & if\;k_{2}\left(\delta_{n}^{3/2}-\delta_{0}^{3/2}\right)\geq k_{1}\delta_{n}^{3/2}\\ F_{0}+k_{2}^{*}\left(\delta_{n}^{3/2}-\delta_{0}^{3/2}\right)+\left(\beta vA_{ij}\right)P_{ij} & if\;k_{1}\delta_{n}^{3/2}>k_{2}^{*}\left(\delta_{n}^{3/2}-\delta_{0}^{3/2}\right)>-k_{c}\delta_{n}^{3/2}\\ F_{0}-k_{c}\delta_{n}^{3/2}+\left(\beta vA_{ij}\right)P_{ij} & if\;-k_{c}\delta_{n}^{3/2}\geq k_{2}^{*}\left(\delta_{n}^{3/2}-\delta_{0}^{3/2}\right) \end{array}\right.$$

where $F_{0}$ is the constant pull-off force, $k_{1}$ is the loading branch stiffness, $k_{2}$ is the loading–unloading branch stiffness, $k_{c}$ is the adhesion branch stiffness, and $\delta_{0}$ is the plastic overlap (deformation). We should stress that $k_{1}$, which is the loading branch stiffness, can be a user-defined parameter or can be calculated based on the Young’s modulus $k_{1}=\frac{4}{3}E^{*}\sqrt{R^{*}}$, and $k_{1}$ is identical to the stiffness for the Hertz contact theory (Section 2.1). Additionally, Equation (6) takes into account the effect of multiple contact points between particles, and it includes four variables: β, ν, $A_{ij}$, and $P_{ij}$. β is a dimensionless empirical prefactor that indirectly accounts for changes in particle geometry. ν is Poisson’s ratio, which reflects a material’s tendency to deform under stress. $A_{ij}$ represents the contact area where two particles are in contact with each other. The equation also involves the isotropic component of stress, which is represented by $P_{ij}$. $P_{ij}$ is calculated by taking the average of the diagonal components of the stress tensors of particles i and j. Specifically, $P_{ij}$ is equal to one third of the sum of the three diagonal components of the stress tensor for each particle.

The full details of the contact model, as well as how it differs from earlier elastic–plastic contact models [26,27], are given in the study by Giannis et al. [4]. This contact model has been validated and successfully captures the main compressibility characteristic (stress–strain) of the compaction of two pharmaceutical materials (Avicel^®^ PH 200 (FMC BioPolymer) and Pharmacel^®^ 102 (DFE Pharma)). This validation was based on the assumption that particles can be approximated by an ideal spherical shape. However, because real particles have an irregular shape in the vast majority of applications, it is important to investigate the influence of particle shape. This study presents a straightforward implementation of this model to address non-spherical particles.

### 2.3. Bonded Multi-Sphere Model (BMS)

In DEM simulations, the proper modeling of particle shape is critical to accurately predict the particle interactions and macroscopic response of the investigated system. The bonded multi-sphere model is one approach that accounts for particle shape. The relationship between particle motion and the forces acting on each particle is determined using Newton’s equations of motion in this approach. The translational and rotational motion equations of a particle are as follows:

(7) $$m_{i}{\ddot{a}}_{i}=\underset{j}{\sum }\left(F_{nij}+F_{tij}\right)+\underset{j}{\sum }\left(F_{bij}\right)+m_{i}g\;\;\;and\;\;I_{i}{\dot{\omega }}_{i}=\tau_{ij}$$

In comparison to Equation (1) in Section 2.1, the term $F_{bij}$ was added, which represents the bonding force between interacting particles. We should also emphasize that the bonding force acts as a long-range force between interacting particles within a cutoff distance, and no overlap is required for the bonding force to be initiated.

With this approach, the irregular shape of particles can be approximated by a cluster of sub-spheres that are bonded together. The bonds not only provide a holistic connection between the sub-spheres, but also allow for relative displacements when external forces act on them. Thus, particle deformation is the result of the relative displacement of all sub-spheres. The contact forces were calculated in this study using two types of contact models: the bonded model was used for intraparticle interactions (non-contacting particles), and, for contacting particles, the Hertz–Mindlin nonlinear elastic model (Section 2.1) or the multi-contact elastic–plastic model was used (Section 2.2).

The bonded-sphere model, based on the beam theory, was initially developed by Potyondy and Cundall [28] to simulate fracture initiation and development across mineral grains in the rock. Following this line of thought and based on the beam theory, this model was further refined and found some intriguing applications in deformable pinewood chips [29], flexible fibers [23,24], and deformable rubber spheres [7]. In general, there are two basic types of bond models: the spring bond model and the beam bond model. A detailed explanation of the distinctions between these approaches is beyond the scope of this study. However, an interesting study by Chen et al. [30] evaluated and attempted to integrate these two approaches.

### 2.4. Conventional Multi-Sphere Model (CMS)

As an alternative to the bonded multi-sphere model presented in Section 2.3, the conventional multi-sphere (CMS) model can be used. In DEM simulations, the CMS approach, as a common way to simulate complicated particle shapes, has to combine spheres of various diameters to form a stiff body (sphere cluster). Spheres within a rigid body are able to overlap since there are no interparticle forces [31]. The relative position of each sphere is always fixed [32]. By summing up the forces that operate on sub-spheres while in contact with other clusters, the total force acting on the rigid body can be calculated. The total force acting on the rigid body is computed by adding the forces acting on the sub-spheres while in contact with other clusters. The elastic–plastic multi-contact model was used in this study to calculate the contact force between the contacting sub-spheres (Section 2.2). The angular momentum of a complex particle was calculated using the total torque on all spheres in regard to the body’s center of mass [33]. Due to the excessive overlap between the sub-spheres, calculating the particle’s moment of inertia is one of CMS’s challenges. One way to overcome this challenge is to calculate the mass and moment of inertia for each complex particle analytically [34]. In order to address this issue, the Monte Carlo method [34] was used in this study.

## 3. DEM Simulations of the Uniaxial Compression of a Single Rubber Sphere

In this section, several approaches to modeling the deformation behavior of a single rubber sphere are discussed. As a baseline, we compared the result of numerical simulations to the experimental data from Tatara [35] for a rubber sphere compressed between two rigid plates. The rubber sphere was chosen as a relevant material due to its elastic (no hysteretic behavior) nature and its strong Poisson’s effect (large deformation behavior with excessive lateral expansion) to demonstrate the capabilities or limitations of the studied numerical methods that deal with large deformations.

### 3.1. The Bonded Multi-Sphere Approach (BMS) and the Multiple-Particle Finite Element Method (MPFEM)

The classical DEM based on the Hertz theory [25] assumes that only small deformations may occur in a body (spherical shape), while its shape remains constant (body rigidity). On the one hand, with MC-DEM, pseudo deformation is allowed and the large deformation of a body can be captured [20]. However, as with classical DEM, there is no shape alteration with this approach; because it accounts for multiple contacts occurring simultaneously (sum of force contribution of neighboring particles), the overall force experienced by a sphere is higher.

The BMS approach can be used as an alternative to bypass the restrictions of the classical DEM and to allow for body deformability, since connected sub-spheres that are bonded together allow the body to perform translational and rotational motions. Another approach for introducing deformability into a body is to use the multiple-particle finite element method (MPFEM) [36,37], which entails meshing each individual particle with finite elements.

**Figure 1:**
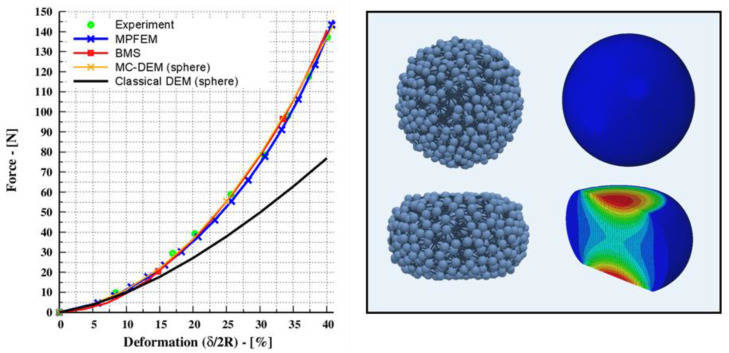
Uniaxial compaction of a rubber sphere. (**Left**): applied force and the resulting deformation, the experimental data from Tatara [35] with deformation of 40%. (**Right**): schematic of spherical representation with BMS (bonded DEM) and MPFEM (FEM).

To illustrate, a single rubber sphere was pressed between two rigid plates, causing up to 40 % of its original diameter to deform ($\frac{\delta }{2R}$). The rubber sphere had a radius of 1.0 cm, and the elastic properties are reported in the work of Giannis et al. [20]. The input parameters are summarized in Table 1. As shown in Figure 1, the rubber sphere was replaced by an agglomerate of the same characteristic diameter that was formed by a number of bonded sub-spheres. In addition, a commercial finite element software suite (ABAQUS^®^ Simulia, Dassault Systèmes, Paris, France), was used to conduct detailed MPFEM simulations. Along with these, MC-DEM and classical DEM were used to study the spherical representations.

The numerical results were then compared to the experimental data provided in Tatara’s original paper [35]. It is worth noting that Tatara’s study explored and demonstrated that the classical Hertz theory is only capable of capturing small deformations (occurring locally) of up to 10% and is not applicable to materials that can endure extensive deformation, such as spherical rubber spheres. As seen in Figure 1, BMS provides very good agreement with experimental data up to the deformation of 40%, and the spherical shape is allowed to deform, resulting in a flat contact area and pressure points. In a similar manner, the MPFEM approach accurately replicates the experimental data, and the spherical shape facilitates lateral expansion; similar results were reported by Agarwal and Gonzalez [38]. On the other hand, classical DEM (with spherical representations) fails after a deformation of 10%. In fact, Dosta et al. [7] presented and verified that the BMS’s upper limit for accurately predicting deformation was 50%. The MC-DEM approach, which is essentially a classical DEM approach with an enhanced contact law, can overcome these limitations and provide a precise description of the experiment results, as shown in Figure 1.

### 3.2. Sensitivity Analysis of BMS and MPFEM Approach

**Figure 2:**
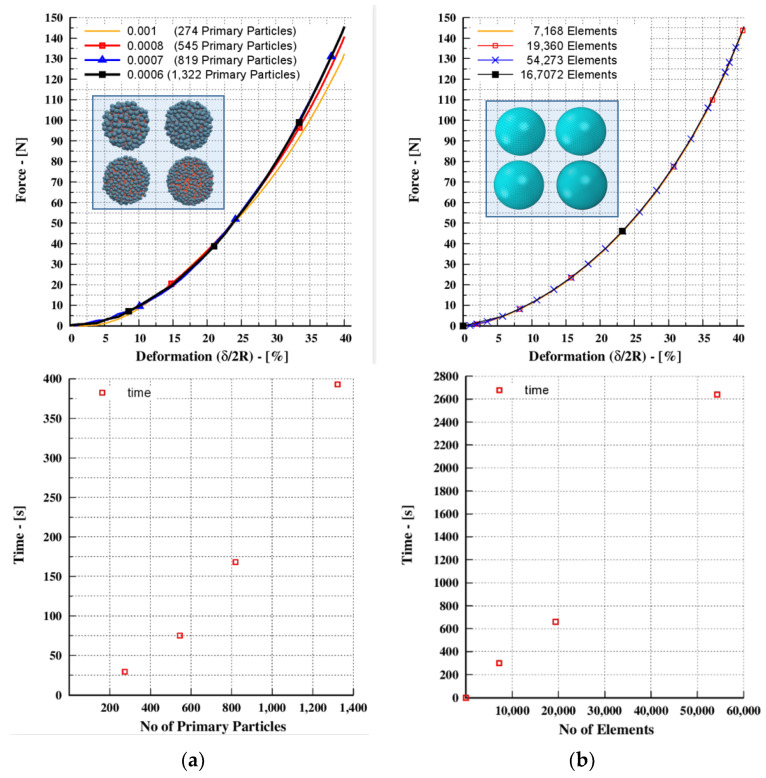
Uniaxial compression of a single rubber sphere: (**a**) force–deformation for BMS and MPFEM approach; (**b**) the computational time depending on the number of particles or elements, respectively.

A sensitivity study was conducted using fine and sparse shape approximations to examine the solution’s stability. As can be seen in Figure 2a, the results of BMS converge as the approximations become finer. For the fine approximations (1322 and 819 sub-spheres), the results are identical, and when the approximations become coarser, the results begin to deviate. This necessitates a sensitivity analysis to determine the optimum size of the approximation that is required. As a rule of thumb, a fine approximation can ensure an accurate solution. However, from the results shown in Figure 2a, it is evident that there is a trade-off between the computational time and approximation size. In other words, it is too expensive in terms of computational time to provide an exact solution and it becomes more expensive when a system with many rubber spheres is examined. To obtain a compromise among the efficiency (in terms of computational time) and accuracy, the representation of 545 primary particles was used for this study.

For the MPFEM approach, the material was described as a compressible neo-Hookean material [38]. Tetrahedral elements were used to discretize the geometry. The sparse and dense meshes are shown in Figure 2b, and the results show that altering the mesh size has no effect on the finite element analysis. For most linear problems, an iterative solution approach is not necessary. However, iteration procedure convergence must also be considered in nonlinear problems [39]. It is expected that the computational time significantly increases because a higher number of elements is needed.

The BMS method and the MPFEM approach are both numerical methods used to simulate the mechanical behavior of materials. However, they differ in their approach to modeling fractures and their computational requirements. One of the main advantages of the BMS method is its ability to handle multiple cracking events and crack propagation naturally. In contrast, the MPFEM approach does not allow for crack propagation and only models cracks as predefined discontinuities in the material. Another difference between the two methods is their computational requirements. This study demonstrated that in the scenario of the uniaxial compression of a single rubber sphere, the BMS method was faster than the MPFEM method in terms of computational time. However, it is important to note that the computational requirements of each method may vary depending on the specific scenario being modeled. Finally, while MPFEM models grains as continuous entities, BMS relies on micro-mechanical parameters [40] and requires numerical calibration to obtain a solution. This means that BMS requires additional calibration, which may increase the complexity of its implementation.

### 3.3. The Conventional Multi-Sphere (CMS)

**Figure 3:**
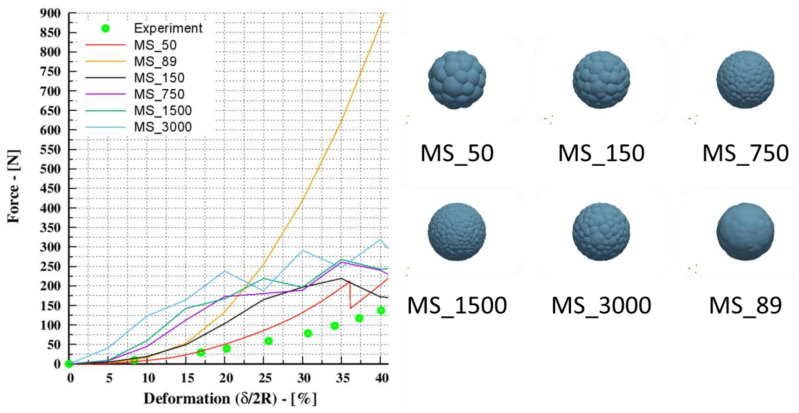
Uniaxial compaction of a rubber sphere. (**Left**): applied force and the resulting deformation, the experimental data from Tatara [35] with deformation of 40%. (**Right**): schematic of spherical representations comprising 50, 89, 150, 750, 1500, and 3000 multi-spheres.

The conventional multi-sphere (CMS) inscribes spherical entities into complicated geometries; the spheres can take an arbitrary position, have a wide range of sizes, and even overlap, resulting in multiple contacts. To illustrate the effect of shape approximation on contact force evolution, this section examines the deformation behavior of a single rubber sphere in a simulation model that is identical to the one discussed in Section 3 and Section 3.1. The sphere was approximated using a multi-sphere approach with various representations that are depicted in Figure 3. The representations comprise 50, 89, 150, 750, 1500, and 3000 multi-spheres. It should be noted that the multi-sphere approach has certain limitations, such as bumpiness, which results in the artificial roughness of the particle [11]. To overcome this, a depiction with a nearly smooth surface was adopted (MS_89 on the right side of Figure 3). The resulting evolution of the contact force was compared to the results of the experimental data (identical to Section 3.1), which acted as a calibration curve.

The results shown in Figure 3 reveal that there is a severe divergence in the resulting force when compared to the outcomes of the various multi-sphere representations. In brief, the results do not converge, which indicates that a different representation would provide a different force vs. deformation curve. The associated curves have irregular dropouts, showing that the number of contact points fluctuates during the compression, and similar results have been reported by Höhner et al. [11]. This can be explained by the fact that the multi-spheres are allowed to overlap and different multiple contact points can occur that contribute to the contact force resolution [11]. To address these limitations, Höhner et al. [11] suggested a calculation procedure to account for the influence of multiple contacts in contact force calculations; however, applying the suggested calculation steps falls beyond the scope of this study.

## 4. Realistic Particle Modeling with the CMS and BMS Approach

Real particles are irregular in shape. However, a typical assumption in the majority of DEM simulations is that the particle’s real shape may be substituted with an ideal spherical one; this is a widespread practice to avoid the additional computational time required for contact detection and resolution when the real shape is considered. As a result, non-spherical representations are seldom used in DEM simulations, and because contact detection and resolution are straightforward, the vast majority of DEM models are designed for systems with spherical particles. In this study, we attempted to investigate how the particle shape affects the macroscopic stress–strain response of the investigated system. The BMS and CMS were used in conjunction with the multi-contact model described in Section 2.2 to accomplish this goal. One method to construct a realistic non-spherical particle representation is by applying X-ray microtomography, where image processing techniques are used to identify the particle shape [41]. Another strategy employed in this work is the generation of random 3D particles that strive to fit the desired shape parameters, such as the average aspect ratio, elongation, etc. The aspect ratio is defined as the ratio of the smallest Feret diameter compared to its largest diameter [42]:

(8) $$ASR=\frac{S}{L}$$

**Figure 4:**
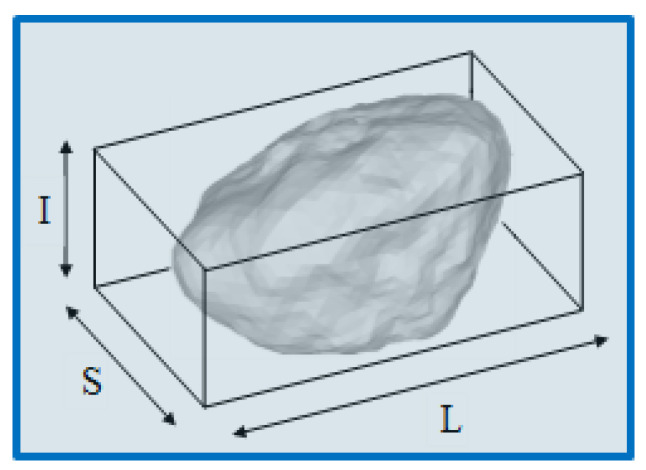
The dimensions of the box enclosing the particles used to define the shape parameters.

In addition to the aspect ratio, the particle shape is characterized by the following parameters [43,44]:

(9) $$elongation=\frac{I}{L},\;\;flatness=\frac{S}{I}$$

where L, I and S are the longest, intermediate, and smallest sides of the box, respectively. A schematic representation is shown in Figure 4.

**Figure 5:**
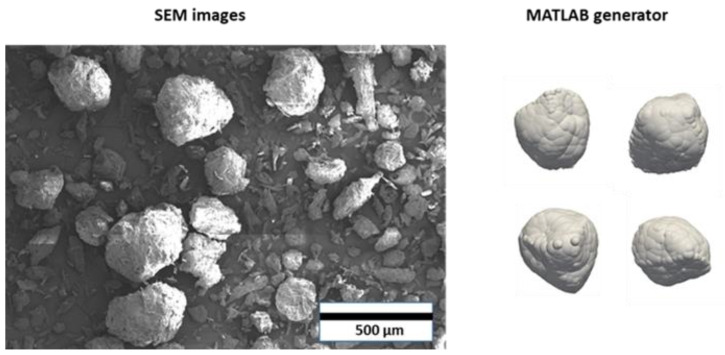
The process of generating realistic non-spherical particles including: SEM images of Avicel^®^ PH 200; MATLAB code generation and filling the multi-sphere.

Another method for quantifying the particle shape is to utilize the closed-form Fourier function, which mathematically characterizes any arbitrary cross-sectional 2D image. To construct virtual complex-shaped particles and examine their packing behavior in defined locations, an unique Fourier–Voronoi-based technique was developed by Mollon and Zhao [44] The authors published a MATLAB algorithm that took in the required Fourier descriptors and generated the particle linked to them. In this study, this algorithm was used to generate non-spherical particles. The process of representing non-spherical particles is shown in Figure 5 and includes two main steps: (a) images of raw materials were taken with the Helios G4 CX DualBeam (Thermo Scientific, Waltham, MA, USA) scanning electron microscope (SEM); (b) the MATLAB generator was used to generate non-spherical particles and fill them with overlapping spheres.

**Figure 6:**
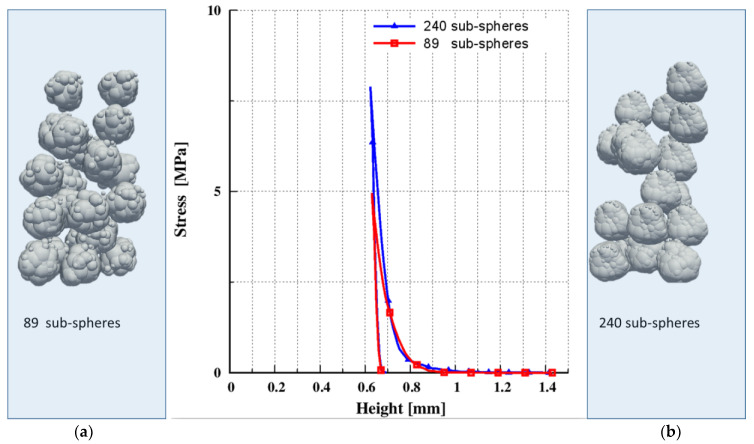
Uniaxial compression of particles with different qualities in approximation and stress–height response: (**a**) based on 89 sub-spheres; (**b**) based on 240 sub-spheres.

The average shape characteristics of the generated four distinct non-sphercial shapes (Figure 5) are ASR=0.8, elongation=0.84, flatness=0.95. The quality of an approximation is one of the most essential challenges for this procedure. In other words, how many overlapping spheres should the system have? A sensitivity analysis with varied numbers of sub-spheres was performed to answer this question. Since the computational cost is high and fine multi-sphere representation requires a large number of particles, only two different approximations were examined, as shown in Figure 6.

### 4.1. Materials

The material studied is a type of microcrystalline cellulose known as Avicel^®^ PH 200 (FMC BioPolymer, Philadelphia, PA, USA). The powder characteristics, including particle size distribution (PSD) and true density, are given in Table 2 and have been previously published in the literature.

### 4.2. Experimental Methods

In order to study how the powder behaves when compressed, in this study, the compaction simulator called Styl’One Evolution (CS; Medel’Pharm, Beynost, France) was used. This device is designed to precisely control the compaction process and collect data on the force and displacement of the powder. The in-die data were analyzed using the ANALIS software from the same manufacturer. The compaction process was carried out using a generic profile that aimed to achieve a compression stress of approximately 30 MPa.

### 4.3. Numerical Example with the CMS Approach

In this section, the uniaxial compression of non-spherical particles is shown. The system under consideration is a cube with dimensions of $1\times 1\times \left(0.5+height\right)\;\mathrm{mm}$ along the x-y-z directions, and while the x and y axes and the bottom of the z direction were fixed in size, the height was left open, since the filling height was investigated. The particles were generated with a random orientation over the whole volume of the cube. Gravitational forces led the particles to settle to the bottom of the cube. Periodic boundaries were used along the x and y axes and the cube contained a top and a bottom plate. A monodisperse system with a characteristic particle diameter (max diameter) of 24 μm was examined in this study—namely, the microcrystalline cellulose-grade Avicel^®^ PH 200. In line with prior simulations using spherical particles, the material and contact model input properties were identical to those reported in the study by Giannis et al. [4] and are given in Table 3.

Following the initial filling, the sample was compressed uniaxially along the z axis from the maximum initial height to the minimum compression height of 0.6 mm, and then decompressed. From the results shown in Figure 6, it is clear that the quality of the approximation had an impact on the results. We can draw the same conclusion as in Section 3.3: the results do not converge on a solution; hence, the CMS was not further employed in this study.

### 4.4. Modeling Compaction of Avicel^®^ PH 200 with BMS Approach

**Figure 7:**
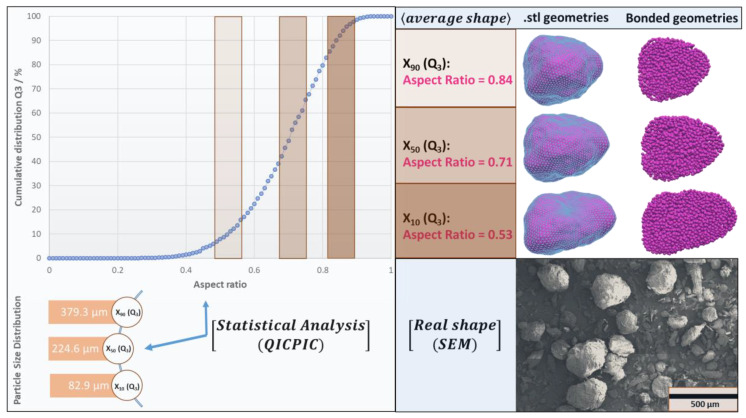
The process of generating realistic non-spherical particles includes statistical analysis with QICPIC; SEM images of Avicel^®^ PH 200; stl geometries that were filled with a number of sub-spheres for BMS DEM simulations.

In order to compare the outcome with the BMS approach that used non-spherical particles, we used the compaction of Avicel^®^ PH 200, as well as the corresponding experimental and simulated data, based on spherical representations from our previous work [4]. Giannis et al. demonstrated the existence of a representative volume element in their work by investigating a number of differently sized representative volume elements (RVEs) and demonstrating that it could be used for DEM simulations to accurately predict the experimental data in this investigation; because there is a great requirement to reduce the computing cost, the smallest of these RVEs was used. In DEM simulations, it is common to use coarse-grained particles with a large diameter to increase the simulation speed [45]. Another way to accelerate the simulations is to establish a minimum diameter size for the particles [46]. In this study, the system under consideration was filled with 150 particles, as shown in Figure 7, with a mean diameter of 225 μm. In order to reduce the computational time of DEM simulations, particles with sizes smaller than 180 μm (a mean diameter of ≈225 μm, Table 2) were excluded. This was done because smaller particles need smaller timesteps, and neglecting them eliminates the need for extremely small timesteps, which would otherwise be necessary. 

To account for the particle shape, a statistical analysis was conducted by dynamic image analysis (QicPic, Sympatec GmbH, Germany).

The cumulative distribution of the aspect ratio (see Figure 7) reveals that the aspect ratio of the particles ranged from 0.4 to 0.9, with a mean aspect ratio of $S_{50}\approx 0.71$. Later, and in line with this information (distribution of the aspect ratio), three non-spherical representations of the particles were generated with the MATLAB generator with respect to the particle size distribution. In the end, the geometries (.stl files) were filled with sub-spheres that could be used by the BMS approach. Represented geometries were used to construct the packing of non-spherical particles. After filling, the particles were relaxed due to the action of gravitational forces. The initial packing density for spherical representations was 59%; to reach this packing density for non-spherical representations, the system had to be compressed. The system was then compressed to a maximum target strain of 57%, before it was decompressed. Three distinct approximations with varied aspect ratios were used for the BMS method, as shown in Figure 7 (0.84, 0.71, 0.53), and to reduce the computational cost, 150 coarse approximations with an average of 350 sub-spheres were used; see Figure 7. A bond is formed between two particles, i and j, with radii $r_{i}$ and $r_{j}$ and positions $x_{i}$ and $x_{j}$, if the distance between the two particles meets the following condition:

(10) $$\left(x_{i}-x_{j}\right)\cdot n<\left(r_{i}+r_{j}\right)\cdot m_{b}$$

where $n=\frac{x_{i}-x_{j}}{\left|x_{i}-x_{j}\right|}$ is the unit vector pointing from particle j to particle i, and $m_{b}$ is a user-defined multiplier that controls the number of bonds that are created at a given timestep.

At the beginning of the simulation, the particles were immediately bonded together during the initial timestep. The multiplier $\left(m_{b}=1.15\right)$ used was carefully selected to ensure that every particle could form bonds with its adjacent particles, and no particle would remain unbonded.

**Figure 8:**
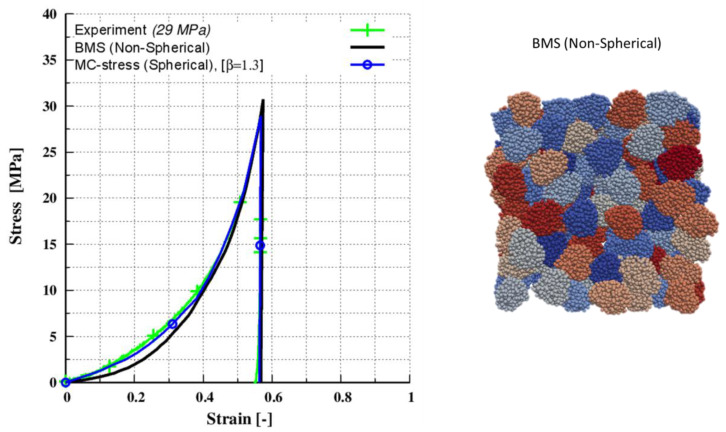
Calibration of Avicel^®^ PH 200 under uniaxial compaction and at maximum target strain of 57%.

A system containing spherical particles, as previously studied by Giannis et al. [4], was used as a reference. For the input parameters given in Table 4 of the BMS method, a calibration strategy including a series of simulations was conducted. As seen in Figure 8, good agreement between the simulated and experimental data was achieved. It should also be noted that this was only possible since the loading stiffness was not dependent on the Young’s modulus. In a future study, a contact model that takes this input as a calibrated parameter (depending on the surface energy, yield strength, and modulus of elasticity) should be investigated.

At this point, the differences between the BMS method and the CMS approach should be explained. The complex particle shape with CMS can be easily captured due to overlapping spheres. However, as shown in this study and in previous studies [33,47] (Kruggel-Emden et al. 2008; Kozhar et al. 2015), the macroscopic response of a multi-sphere particle is determined by the quality of shape approximation. As such, different shape approximations yield different results. Alternatively, with the BMS method, the particle’s real shape was captured by the sub-spheres; an investigation to determine the size of the sub-spheres was needed, as they are glued together with solid bonds. With this came the necessity to calibrate the bond input parameters, whereas the number of overlapping spheres was necessary with the CMS approach. This weakness, however, was also a strength for the BMS, since the bonds would be tuned accordingly, allowing for flexible particle behavior (Section 3.1, Section 3.2 and Section 4.2) or, if necessary, the study of breaking or crushing events. The particle in CMS behaves similarly to a rigid body; therefore, large deformation (Section 3.2 and Section 4.1) is not yet achievable. Nevertheless, breaking or crushing events can be achieved by the immediate release of the sub-spheres after an energy threshold is reached.

## 5. Conclusions

In this study, the compression behavior of a single rubber sphere was studied using various numerical techniques: BMS and CMS. The results were compared to experimental data and detailed finite element simulation (MPFEM), and it was found that BMS method accurately reproduced the experimental data. However, the CMS method did not converge to a solution, even when using different numbers of sub-sphere approximations. This is because the CMS method allows several spheres to overlap, resulting in numerous contact points that contribute to the resolution of the contact force. We also demonstrated that the bonded multi-sphere (BMS) naturally handles large deformations and allows the particle to alter its shape, whereas, using the conventional multi-sphere (CMS), the particle remains rigid. As a result, the BMS method was applied using realistic particles to investigate the compaction behavior of Avicel^®^ PH 200 and, in conjunction with multi-contact DEM, very good agreement with the experimental data was achieved. The results were then compared with those from MC-DEM, which only employs spherical particles. The comparison revealed that the assumptions made about spherical representations were valid and could lead to precise results.

The methodologies investigated in this study have a significant drawback in terms of high computational time, which limits the number of particles that can be simulated. Consequently, future research should focus on utilizing surrogate models to accelerate the simulations. This would be crucial in enabling larger-scale simulations and making the computational modeling approach more efficient.

## Figures and Tables

**Table 1:** Input parameters used for simulations.

Parameter	Symbol	Values	Units
Young’s modulus—particle (p)	*E*	18.5	MPa
Young’s modulus—particle (w)	*E*	1	GPa
Particle density	*ρ*	2000	kg/m^3^
Poisson’s ratio	ν	0.46	-
Coefficient of restitution	*COR*	0.7	-
Friction coefficient	*f*	0.5	-
Bond normal stiffness	$k_{n}^{b}$	$1.15\times 10^{6}$	N/m^3^
Bond tangential stiffness	$k_{t}^{b}$	$1.25\times 10^{6}$	N/m^3^
Empirical prefactor	*β*	1.71	-

**Table 2:** Particle size distribution (PSD) and true density [4].

Material	$x_{10}\left(Q_{3}\right)$ $\left(\mu m\right)$	$x_{50}\left(Q_{3}\right)$ $\left(\mu m\right)$	$x_{90}\left(Q_{3}\right)$ $\left(\mu m\right)$	Span (-)	$\mathrm{True}\;\mathrm{Density}\;(kgm^{-3})$
Avicel^®^ PH 200	82.9	224.6	379.3	1.32	1541.1

**Table 3:** The input parameters for particle–particle and particle–wall interactions.

Property	Symbol	Values	Units
Young’s modulus—particle (p)	*E*	$2.58\times 10^{8}$	MPa
Young’s modulus—wall (w)	*E*	$7.62\times 10^{10}$	MPa
Poisson’s ratio—particle	*ν*	0.30	-
Poisson’s ratio—wall	*ν*	0.31	-
Coefficient of restitution particle	*COR (p-p)*	0.352	-
Coefficient of restitutio—wall	*COR (p-w)*	0.352	-
Coefficient of sliding friction—(*p-p*)	μ_s*(pp)*_	0.561	-
Coefficient of sliding friction—(*p-w*)	μ_s*(pw)*_	0.707	-
Coefficient of rolling friction—(*p-p*)	μ_r*(pp)*_	0.3	-
Coefficient of rolling friction—(*p-w*)	μ_r*(pp)*_	0.01	-
Density	*ρ*	1541.1	kg/m^3^

**Table 4:** The input parameters used in the BMS approach.

Property	Symbol	Values	Units
Young’s modulus—particle(p)	*E*	$2.58\times 10^{8}$	MPa
Young’s modulus—wall(w)	*E*	$7.62\times 10^{10}$	MPa
Poisson’s ratio—particle	*ν*	0.30	-
Poisson’s ratio—wall	*ν*	0.31	-
Coefficient of restitution particle	*COR (p-p)*	0.352	-
Coefficient of restitutio—wall	*COR (p-w)*	0.352	-
Coefficient of sliding friction—(*p-p)*	μ_s*(pp)*_	0.561	-
Coefficient of sliding friction—(*p-w*)	μ_s(*pw)*_	0.707	-
Coefficient of rolling friction—(*p-p*)	μ_r*(pp*)_	0.3	-
Coefficient of rolling friction—(*p-w*)	μ_r*(pp)*_	0.01	-
Density	*ρ*	1541.1	kg/m^3^
Bond normal stiffness	$k_{n}^{b}$	$8\times 10^{8}$	N/m^3^
Bond tangential stiffness	$k_{t}^{b}$	$9\times 10^{8}$	N/m^3^
Empirical prefactor	*β*	1.3	-

## Data Availability

Not applicable.
